# The Simplest Method for Fishhook Removal: The String-Yank Technique

**DOI:** 10.7759/cureus.73885

**Published:** 2024-11-17

**Authors:** Takashi Watari

**Affiliations:** 1 Integrated Clinical Education Center, Kyoto University Hospital, Kyoto, JPN; 2 General Medicine Center, Shimane University Hospital, Izumo, JPN

**Keywords:** fish hook removal, fishing injuries, minor surgery, sport injury, string-yank technique

## Abstract

Fishing is a popular recreational activity worldwide, but complications arise when fishhooks become embedded in the skin. Barbed hooks, due to their design, are particularly challenging to remove. While many individuals attempt self-extraction, some present to emergency departments for medical intervention. Several techniques, including the push-through and needle methods, are commonly employed for hook removal. The String-Yank technique, a simple yet effective approach, is frequently overlooked in clinical settings despite its wide use among fishermen. This case report demonstrates the string-yank technique in a 42-year-old male patient who presented with a 1.5-cm barbed hook embedded in his right little finger. After local anesthesia with lidocaine, the string-yank technique was performed, allowing for the rapid, painless removal of the hook without complications. Post-procedural care included wound disinfection and infection prevention counseling. The string-yank technique offers a minimally invasive, low-pain option for fishhook removal, particularly in superficial injuries. However, its application may be limited in cases of complex injuries or larger hooks. This report aims to provide a clear, step-by-step guide for this underutilized method, emphasizing its benefits in emergency care.

## Introduction

Fishing is one of the most popular recreational activities worldwide. Unfortunate events can occur when the fish hook becomes embedded in the body. In many cases, individuals attempt to remove the hook themselves, often avoiding medical consultation. However, in more challenging situations, patients visit emergency rooms or clinics. Barbed hooks are particularly difficult to remove owing to their design [1]. Methods such as the push-through technique [1,2], which involves pushing the hook through the skin and cutting off the barb, and the needle technique [1,2], which uses an 18-gauge needle to cover the barb, are commonly used. However, the string-yank technique, which is a simple yet effective method, can quickly remove most fishhooks while minimizing pain [3,4]. Despite its popularity among fishermen, there are few detailed video reports on this technique [3,4]. This study aimed to provide a step-by-step guide for the string-yank technique using real patients' video lectures suitable for emergency care situations.

## Case presentation

A 42-year-old male patient with no significant medical history, medication use, or allergies presented to the clinic at 6 AM with a fishhook embedded in his right little finger while fishing. The hook was a 1.5-cm barbed hook. Physical examination revealed that the hook was embedded at an approximately 60-degree angle, with the barb completely buried. There was no evidence of inflammation or neurovascular injury. The patient successfully had the fishhook removed using the String-Yank technique without any complications (Video 1).

**Video 1:** String-yank technique This video explains the step-by-step procedure for safely removing a fish hook from the skin, emphasizing the preparation, proper technique, and aftercare.

The patient recovered completely within a few days without any complications. Subsequently, the author explains the detailed procedure using a step-by-step video demonstration.

## Discussion

The key points to which special attention for the string-yank technique are as follows.

Procedure Steps

Preparation: The affected area was disinfected, and lidocaine was applied to the entry site. While waiting for the anesthetic to take effect [5], the procedure was explained, and the patient was reassured. Clear communication and empathy are crucial, as patients often fear the hook being forcefully pulled out. Practitioners should wear protective glasses to avoid injury from the recoiling hook.

Setting the string and preparing for removal: A strong string was wrapped around the shank of the hook and secured in the dominant hand. Stabilizing the hook shaft against the skin is key.

Removal: The string was pulled swiftly and firmly in the direction opposite to the hook’s entry while maintaining pressure on the shaft. The hook was removed cleanly in one motion.

Aftercare: The wound was disinfected, and the patient was instructed on infection prevention and advised to monitor for signs of redness, swelling, or drainage. Proper education on aftercare is vital.

One retrospective study with 165 patients indicated that approximately 80% of fish hook injuries occurred to the hand, and the "advance and cut method" was the most common extraction method (56%). [6] However, the String-Yank method offers a quicker, less invasive alternative that typically results in smaller scars, reduced pain, and higher patient satisfaction. In this case, despite the barb being completely embedded, the hook was removed swiftly and effectively. Although the string-yank method is advantageous, its effectiveness may vary depending on the type of hook and the anatomical location of the injury. Some studies have suggested that other methods may be preferable, especially for complex injuries, such as multiple sutures with double hooks, treble hooks, eye punctures, and large-sized fish hooks [1,6]. However, it may result in higher postoperative complications when used for complex hooks, highlighting the need for quantitative studies to systematically track complications and evaluate wound healing. Overall, the string-yank technique is a promising approach for managing fish hook injuries, balancing efficacy and safety for superficial skin hook trauma.

## Conclusions

In this video report, the string-yank technique is clearly explained in a manner that can be easily understood and performed by beginners. The string-yank technique is a valuable first-line approach for removing embedded fish hooks and should be widely used in emergency settings. However, alternative methods should be considered, depending on the location of the injury, depth, and hook size.
